# Machine learning models for predicting risks of MACEs for myocardial infarction patients with different VEGFR2 genotypes

**DOI:** 10.3389/fmed.2024.1452239

**Published:** 2024-09-05

**Authors:** Alexander Kirdeev, Konstantin Burkin, Anton Vorobev, Elena Zbirovskaya, Galina Lifshits, Konstantin Nikolaev, Elena Zelenskaya, Maxim Donnikov, Lyudmila Kovalenko, Irina Urvantseva, Maria Poptsova

**Affiliations:** ^1^Faculty of Computer Science, AI and Digital Science Institute, International Laboratory of Bioinformatics, Higher School of Economics University, Moscow, Russia; ^2^Department of Cardiology, Surgut State University, Surgut, Russia; ^3^Institute of Chemical Biology and Fundamental Medicine, Novosibirsk, Russia; ^4^Federal Research Center Institute of Cytology and Genetics, Novosibirsk, Russia; ^5^Department of General Pathology and Pathophysiology, Surgut State University, Surgut, Russia; ^6^Ugra Center for Diagnostics and Cardiovascular Surgery, Surgut, Russia

**Keywords:** machine learning, myocardial infarction, vascular endothelial growth factor receptor 2 gene, statins, prognostic modeling, MACEs, left ventricular wall

## Abstract

**Background:**

The development of prognostic models for the identification of high-risk myocardial infarction (MI) patients is a crucial step toward personalized medicine. Genetic factors are known to be associated with an increased risk of cardiovascular diseases; however, little is known about whether they can be used to predict major adverse cardiac events (MACEs) for MI patients. This study aimed to build a machine learning (ML) model to predict MACEs in MI patients based on clinical, imaging, laboratory, and genetic features and to assess the influence of genetics on the prognostic power of the model.

**Methods:**

We analyzed the data from 218 MI patients admitted to the emergency department at the Surgut District Center for Diagnostics and Cardiovascular Surgery, Russia. Upon admission, standard clinical measurements and imaging data were collected for each patient. Additionally, patients were genotyped for VEGFR-2 variation rs2305948 (C/C, C/T, T/T genotypes with T being the minor risk allele). The study included a 9-year follow-up period during which major ischemic events were recorded. We trained and evaluated various ML models, including Gradient Boosting, Random Forest, Logistic Regression, and AutoML. For feature importance analysis, we applied the sequential feature selection (SFS) and Shapley’s scheme of additive explanation (SHAP) methods.

**Results:**

The CatBoost algorithm, with features selected using the SFS method, showed the best performance on the test cohort, achieving a ROC AUC of 0.813. Feature importance analysis identified the dose of statins as the most important factor, with the VEGFR-2 genotype among the top 5. The other important features are coronary artery lesions (coronary artery stenoses ≥70%), left ventricular (LV) parameters such as lateral LV wall and LV mass, diabetes, type of revascularization (CABG or PCI), and age. We also showed that contributions are additive and that high risk can be determined by cumulative negative effects from different prognostic factors.

**Conclusion:**

Our ML-based approach demonstrated that the VEGFR-2 genotype is associated with an increased risk of MACEs in MI patients. However, the risk can be significantly reduced by high-dose statins and positive factors such as the absence of coronary artery lesions, absence of diabetes, and younger age.

## 1 Introduction

Despite significant advances in cardiovascular surgery, interventional procedures, and pharmacotherapy, the in-hospital and long-term rates of major adverse cardiac events (MACEs)—including death, recurrent acute coronary syndrome (ACS), stroke, and myocardial revascularization—remain high worldwide among myocardial infarction (MI) patients (1, 2).

Secondary prevention programs for these individuals are based on identification of high-risk patients using models that focus on clinical features of MI (ST-segment deviation, left ventricular ejection fraction (LVEF), Killip class, and coronary artery lesions) and conventional risk factors (hypertension, diabetes, chronic kidney disease, dyslipidemia, and smoking) that worsen cardiac outcomes (3, 4).

Identifying patients at high risk of MACEs is crucial. Various clinical scores, such as GRACE, ACEF, CADILLAC, TIMI, PROCAM, PREDICT, PURSUIT, DAPT, and PRECISE-DAPT are available, These scores, primarily based on regression models using clinical parameters, estimate cardiovascular (CV) risk over a 1- to 3-year period after an index MI. Nevertheless, modern personalized approaches to the treatment of post-MI individuals already require more precise clinical scoring systems incorporating biomarkers and genetics data (5, 6).

The pathological basis of coronary artery disease (CAD) and acute coronary syndrome (ACS) is atherosclerosis. Factors such as lipid metabolism disorders, vascular endothelial cell damage, inflammation, and immune dysfunction can promote the development and progression of coronary atherosclerosis, potentially leading to CAD and ACS. The vascular endothelial growth factor (VEGF) family is involved in angiogenesis, inflammation, oxidative stress, and lipid metabolism, presenting potential therapeutic and prognostic value for ACS and MI (7, 8).

The prognostic performance of VEGF serum levels following cardiac ischemia shortly after MI was previously investigated in the Coronary Disease Cohort Study. VEGFs primarily bind to three tyrosine kinase receptors (VEGFR-1, VEGFR-2, and VEGFR-3) with different affinities. Single nucleotide polymorphisms (SNPs) from VEGFR-2 are associated with the development of cardiovascular disease (7). Moreover, three SNPs of the VEGFR-2 gene were identified and significantly associated with coronary atherosclerosis: +1192C > T (rs2305948), −604 T > C (rs2071559), and + 1719A > T (rs1870377) (9–13). Consequently, VEGFR-2 gene allelic variants have the potential to be evaluated as prognostic markers in MI (14).

Recently, machine learning (ML) approaches have proven efficient in predicting patient outcomes based on clinical, imaging, and biomarker data. The largest study, based on the clinical data including 23,000 patients in the BleeMACS and RENAMI registries, showed that ML models can predict all-cause death, recurrent MI, and major bleeding after ACS (15) using 25 clinical features. Other approaches, based on considerably smaller datasets, have also proven the efficacy of ML models for cardiovascular outcomes research (16–20). One advantage of ML modeling is its ability to perform feature importance analysis and rank features according to their contribution to model performance. This not only reveals the most important features but also helps to select a minimal set that can reduce overfitting and improve the accuracy of the model. Additionally, an ML model with a small set of features can be easily applied in practice. Thus, ML models have shown that serum creatinine and LVEF alone can predict all-cause death (16). In another study (21), an ML model trained to predict all-cause death reduced the feature space from 430 to 25. Other models provide evidence of the predictive power of ML models and present the most important features. These include models for prognosis of post-thrombotic syndrome (22), prognostication of the time to death of patients in the Coronary Care Unit (23), prediction of mortality and heart failure (HF) hospitalization in patients with preserved LVEF (17), prediction of long-term risk of MI and cardiac death (24), and in-hospital all-cause mortality in HF patients (20). All the aforementioned ML models were trained using clinical features routinely measured at hospital admission. In some studies, these features were complemented by demographic and social characteristics.

All proposed prognostic ML models do not have high-performance metrics with ROC AUC lying in the interval of 70–80%. One way to improve ML model prediction power is to include more information about patient status, with genetic markers potentially serving a key role. Adding genetic markers as features to an ML model does not mean that clinical features are not prognostic or that, in the future, they are supposed to be replaced with genetic markers only. However, genetic markers can significantly improve patient risk assessment when taken together with other clinical characteristics. This kind of model is limited due to the high-cost genetic testing, and most MI patient clinical datasets do not contain genetic information. Here, for the first time, we incorporate genetic information into an ML model to predict MACEs in MI patients.

## 2 Methods

### 2.1 Clinical data collection

The prospective observational study consecutively included 218 patients with acute MI who were admitted to the Surgut District Cardiology Clinic of the Center for Diagnostics and Cardiovascular Surgery. This study was approved by the local ethics committee at the clinic. During the initial admission to the emergency department, medical researchers (cardiologists) explained the main points of the study protocol to the patients and obtained their informed consent to participate. Then, the patients were transported to the operating unit to undergo emergent coronary angiography (CAG). Significant lesions (stenoses) in the coronary arteries were considered present when the lumen was narrowed by more than 70%. For the left main coronary artery (LMCA), significant stenosis was considered present when the lumen was narrowed by more than 50%. Based on the CAG results, balloon angioplasty and stenting or coronary artery bypass grafting (CABG) were performed. Standard transthoracic echocardiography was also performed during in-hospital treatment.

All patients received guideline-based therapy, including RAAS-blockers, beta-blockers, statins, and dual antiplatelet therapy. High-dose statin therapy was defined as atorvastatin 40–80 mg and rosuvastatin 20–40 mg daily, whereas low-dose statin therapy was defined as atorvastatin 10–20 mg and rosuvastatin 5–10 mg daily, for at least 12 months post-MI.

The inclusion criteria were as follows: acute myocardial infarction with or without ST-segment elevation and age range of 30–70 years. The exclusion criteria were as follows: contraindications to the use of statins; pregnancy and lactation; advanced tumor disease; HIV; and patient’s refusal to participate in the study.

During the laboratory stage, DNA was isolated from the leukocyte rings in the collected blood samples and then frozen at −80°C for future genetic testing. VEGFR-2 (rs2305948) genotypes (C/C, C/T, T/T) were determined by real-time polymerase chain reaction (PCR) using a Real-Time CFX96 Touch device (Bio-Rad Laboratories, United States). The T-allele of VEGFR-2 (rs2305948) is commonly regarded as a risk allele for unfavorable ischemic post-MI outcomes. Glomerular filtration rate (GFR) was assessed using a CKD-EPI calculator to classify chronic kidney disease. Major ischemic events (cardiovascular death, recurrent ACS, stroke, and myocardial revascularization) were registered based on the results of clinical observation between 9 and years (2015–2024) after the indexed MI. The information was collected from in-hospital medical records of patients who were re-admitted to the clinic after the index MI. Additionally, patients were contacted by phone, and the fact of hospital treatment for ischemic events in other medical organizations was confirmed by requesting corresponding medical records. Cases of death in the post-infarction period were recorded in the health insurance policy database, while cardiovascular (ischemic) causes of death were specified by clinical autopsy services based on the patient’s residence. Basic statistics analysis was performed in the SPSS 23.0 software package.

### 2.2 Data preprocessing

For logistic regression, all non-categorical features were normalized using Standard Scaler. For tree-based models that do not require normalization, data transformation was not performed. There was no additional preprocessing of categorical features as all categorical features were either binary or ordered.

For data imputation, we used the Multivariate Imputation by Chained Equations (MICE) method (25). A Linear Regression model with default parameters was used as an estimator for MICE with 100 iterations for numerical data. For categorical and binary features, we used the logistic regression model with a ‘liblinear’ solver as the default estimator for MICE with 100 iterations.

The dataset was randomly split in a 75:25 ratio into training (*n* = 163) and test (*n* = 55) sets respectively, to develop and validate the models. Data imputation and scaling, hyperparameter tuning, feature selection, and the final model training were performed on the training set. Evaluation of metrics of the developed models was performed on the test set.

### 2.3 Machine learning models

We built machine learning models based on Gradient Boosting (Catboost and LightGBM), Random Forest, and Logistic Regression algorithms with hyperparameter tuning using 10-fold cross-validation on the training dataset. For the AutoML approach, we used FLAML (26), a Python package for automatic machine learning that automates the process of model selection, hyperparameter tuning, and feature construction.

To optimize model performance, we used Optuna (27), a Python package that automates hyperparameter tuning using Bayesian optimization algorithms. We utilized a tree-structured Parzen Estimator with 10 startup trials as our search algorithm and used 10-fold cross-validation during the optimization process to obtain a robust estimate of the ROC AUC metric. The sequential forward selection (SFS) method was used to determine the optimal number of training features. During the feature selection cycle, we used 5-fold cross-validation and used the mean ROC AUC as the evaluation metric. The model with the best average metric was chosen as the baseline model for the feature selection procedure. We added one feature at a time until we explored all the features and then selected the subset with the best metric as the final feature subset.

Feature importance analysis was performed using Shapley’s scheme of additive explanation (SHAP) (28). SHAP provided visualization of the feature importance for the selected model and individual contributions of features to the predictive power of the model. Additionally, we constructed partial dependence plots (PDP) and individual conditional expectation (ICE) (29) plots.

To evaluate the predictive performance of the developed models, we scored data samples from the test set. Specifically, we generated 1,000 bootstrap samples of the initial test sample size to obtain the distribution of PR AUC and ROC AUC. By averaging the metrics across all iterations, we obtained the average PR AUC and ROC AUC as the final evaluation metrics.

## 3 Results

### 3.1 Characteristics of MI patients

The dataset used in this study included 218 patients admitted to the emergency department at the Surgut District Center for Diagnostics and Cardiovascular Surgery, Russia, with MI. For each patient, standard clinical measurements, including laboratory tests and imaging indices were selected for inclusion in the model (Table 1). Additionally, each patient was genotyped for VEGFR-2 variation, rs2305948 which includes the C/C, C/T, and T/T genotypes.

**Table 1:** Clinical and imaging characteristics, laboratory, and genetic testing of patients with MI (*n* = 218).

Characteristics	*N*
**Clinical and imaging characteristics**	
Sex, *n* (%)	
Male	169 (78)
Female	49 (22)
Age, years, M ± SD	58 ± 10
BMI, kg/m^2^, Me [25%; 75%]	29 [26; 33]
ECG ST—segment deviation, *n* (%)	
Elevation	74 (34)
Non-elevation	144 (66)
Infarcted walls of LV, *n* (%)	
Anterior wall	95 (44)
Posterior wall	97 (45)
Lateral wall	79 (36)
Killip class, *n* (%)	
I–II	213 (98)
III–IV	5 (2)
LV EF	
< 40%	16 (7)
≥ 40%	202 (93)
Hypertension, *n* (%)	179 (82)
Diabetes mellitus type 2, *n* (%)	52 (24)
Chronic kidney disease ≥ C2, *n* (%)	74 (34)
Family history of CVD	35 (16)
Stenotic lesions of coronary arteries, *n* (%)	
Left main coronary artery	14 (7)
One vessel	79 (36)
Two vessels	63 (29)
≥ 3 vessels	72 (33)
Duration of angina from the onset of acute MI, *n* (%)	
1–3 h	167 (77)
3–12 h	9 (4)
> 12 h	42 (19)
Myocardial revascularization, *n* (%)	
PCI	207 (95)
CABG	11 (5)
Statins (atorvastatin, rosuvastatin)	
High doses	117 (54)
Low doses	101 (46)
**Laboratory and genetic testing**	
High-sensitive troponin T, ng/L, Me [25%; 75%]	110 [45; 408]
LDL cholesterol, mmol/L, M ± SD	3 ± 1
Hemoglobin, g/L, Me [25%; 75%]	139 [128; 149]
Glucose, mmol/L, Me [25%; 75%]	6 [5; 8]
Creatinine, μmol/L, Me [25%; 75%]	83 [72; 95]
VEGFR-2 genotypes (rs2305948), *n* (%)	
C/C—wild type—homozygous for major allele	142 (65)
C/T—heterozygote (major allele + minor allele)	75 (34)
T/T—homozygous for minor allele	1 (1)

C, cytosine—major allele (wild); T, thymine—minor allele with atherothrombotic risk.

### 3.2 Machine learning models

**Figure 1:**
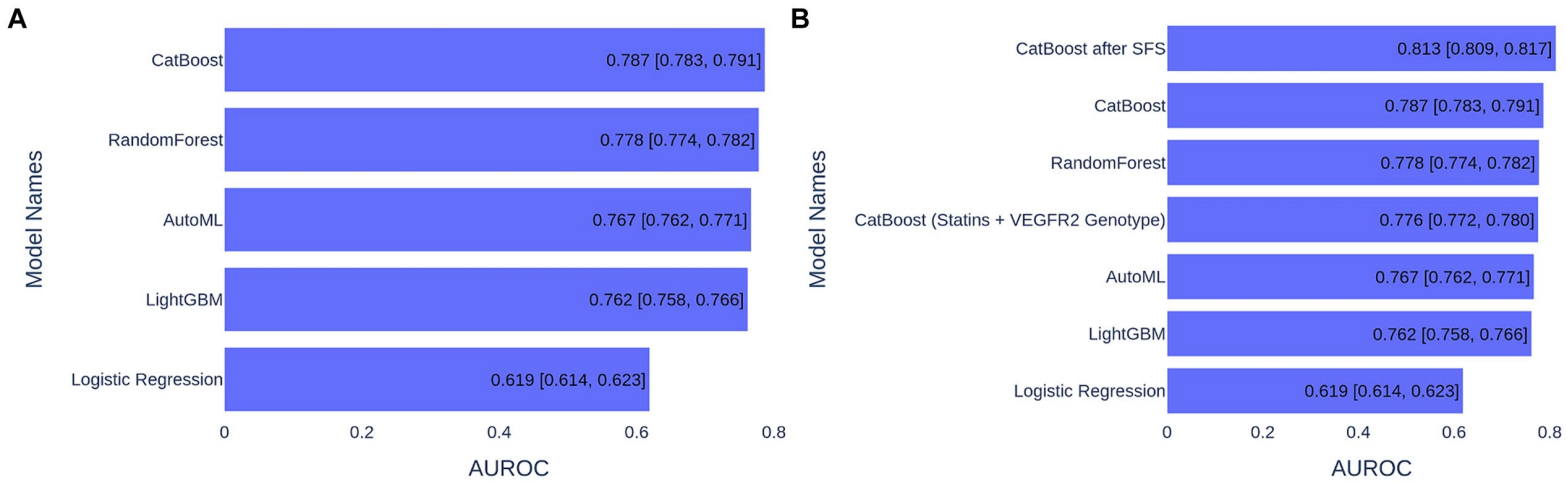
ML model performance comparison based on ROC AUC metrics for 1,000 bootstrapped test sets. **(A)** ML models trained on 39 features. **(B)** ML models trained on various number of features.

The data underwent imputation and preprocessing and were split into training and test sets in a 75–25% ratio for training and validation. In order to choose the best model, we trained and tested the following ML algorithms: Gradient Boosting (CatBoost and LightGBM), Random Forest, Logistic Regression, and AutoML approach (see Methods section for details). For model performance metrics, we took an average ROC AUC calculated on the bootstrap samples from the test set. Model performance comparison is presented in Figure 1A, and the best metric was obtained for the CatBoost model (ROC AUC of 0.787), which was significantly better than the second-best Random Forest (*p* < 1e−03, *t*-test) (Figure 1A). The CatBoost ROC curves for training and test sets are given in Supplementary Figure S1. We chose the CatBoost model for feature selection and feature importance analysis.

### 3.3 Feature selection to find the best model

**Figure 2:**
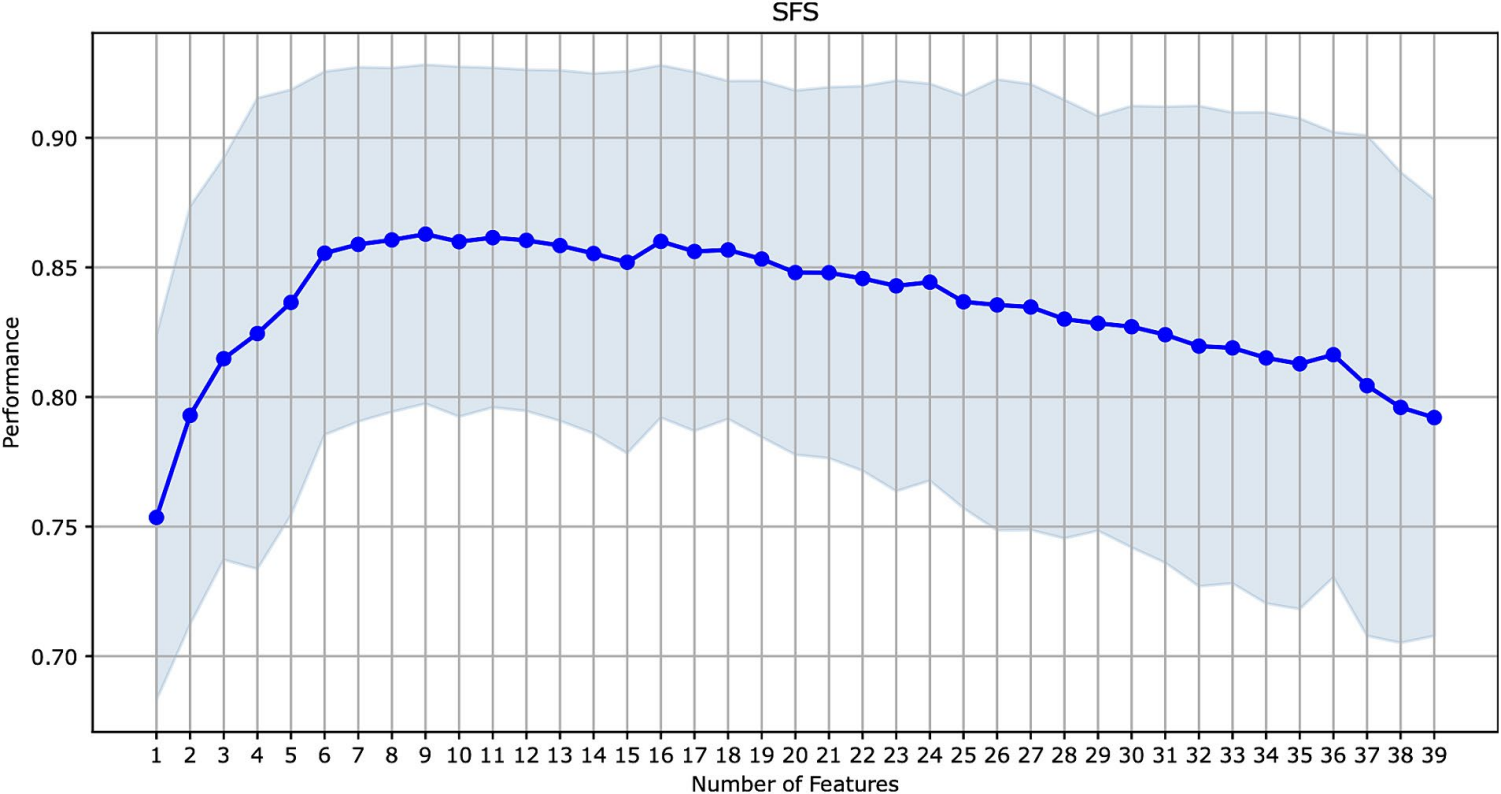
Sequential forward feature selection for the model with the best performance on all features (CatBoost).

The initial ML models were trained on 39 features (Table 2). We explored the contribution of each feature to model performance using the forward sequential feature selection (SFS) method (see Figure 2). We chose the SFS method due to its balance between computational efficiency and robustness. SFS directly evaluates the contribution of each feature to model performance using cross-validation at each step, ensuring a resilient and accurate feature selection process. This avoids potential inaccuracies associated with heuristic methods like feature importance in tree-based methods or feature imputation, which can be misleading, especially with high cardinality features. Thus, SFS is a simple and straightforward method for the selection of a minimal set of features that provide maximal precision of an ML model.

**Table 2:** List of 39 features initially included in the model.

1.	Age
2.	Anterior LV wall
3.	Atrial fibrillation
4.	BMI
5.	BMS or DES
6.	Comorbidity Index
7.	Coronary artery lesion
8.	Creatinine
9.	Diabetes
10.	Duration of pain
11.	Sex
12.	Glucose
13.	Heart rate
14.	Hemoglobin
15.	Family history of CVD
16.	Hypertension
17.	Infarct-related artery
18.	Killip class
19.	LV EDV
20.	LV EF
21.	LV ESV
22.	LV infarct size
23.	LV mass
24.	Lateral LV wall
25.	Left atrium
26.	Left main coronary artery lesion
27.	Multifocal atherosclerosis
28.	PCI or CABG
29.	Post-MI
30.	Post-stent
31.	Post-stroke
32.	ST segment
33.	Statin dosage
34.	Systolic BP
35.	TIMI grade in infarct-related artery
36.	TnT PO
37.	Total cholesterol
38.	VEGFR2 genotype
39.	eGFR EPI

Highlighted are nine features selected by the SFS method.

**Figure 3:**
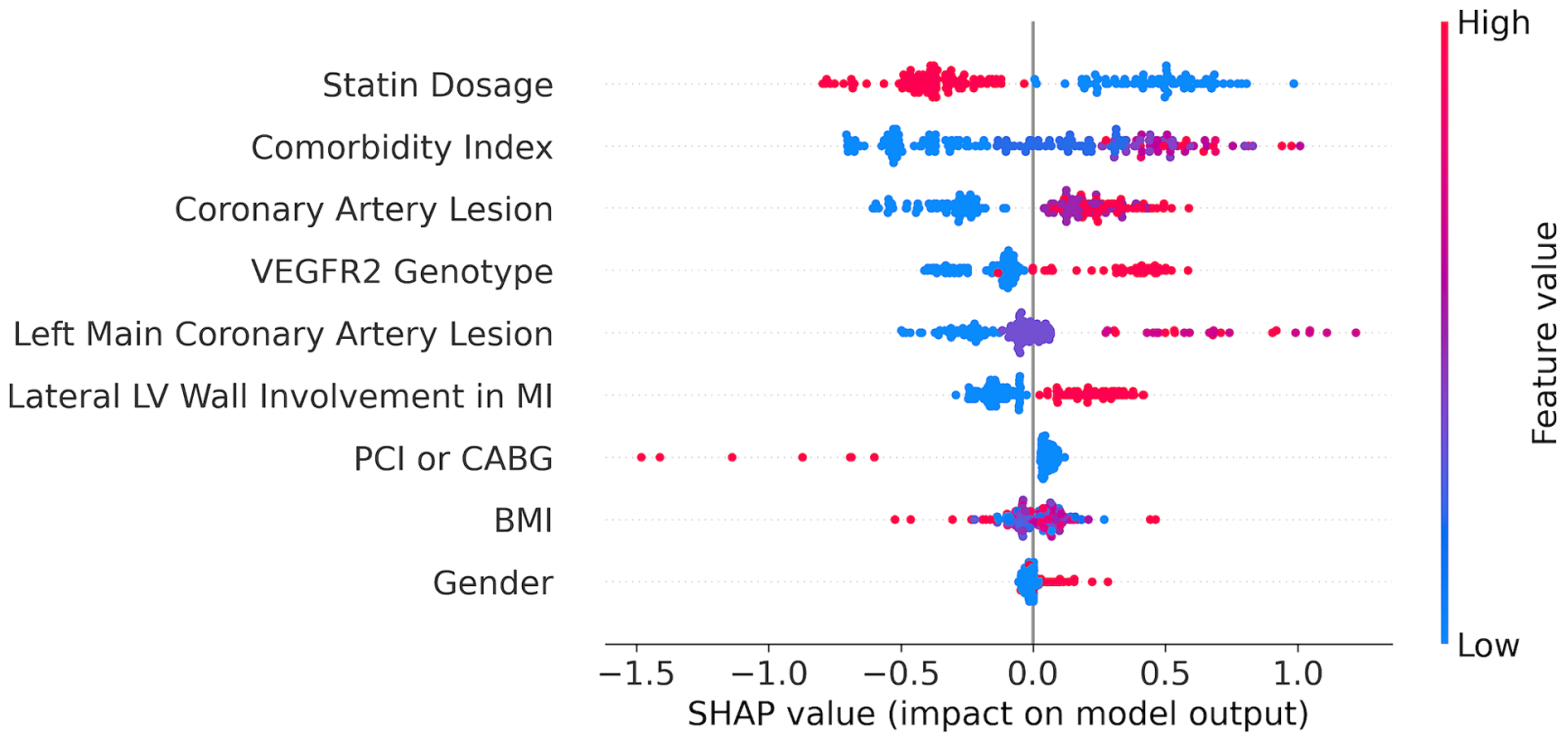
SHAP feature importance plot for Catboost model built on 9 SFS-selected features.

Since CatBoost showed the best performance with 39 features, it was used as the baseline model for SFS. The model’s performance increased gradually with the addition of the first nine features, but then began to decline as additional features were included. These first nine features (presented in Figure 3) were selected for retraining the CatBoost model (Supplementary Table S1). The CatBoost model trained on the nine selected features showed statistically significant improvement in the model’s performance compared to the CatBoost model trained on 39 features (ROC AUC of 0.813 vs. 0.787, *p* = 5e−19, *t*-test) (Figure 1B and Supplementary Figure S2). Thus, the CatBoost model trained on nine features was chosen for further analysis of MI patients.

### 3.4 Feature importance analysis

For feature importance analysis, we chose SHAP because it estimates the contribution of the selected features to the model performance, and it considers the cooperative effect of other features. SHAP values can be added to show the joint contribution of several features to a final ML model prediction. This helps to understand the combined effect of multiple features on risk assessment.

We applied the SHAP method using the best CatBoost model with the nine features selected by the SFS method (Figure 3). The nine features are ranked from the most important (top) to the least important (bottom). A higher SHAP value for a feature indicates a greater risk of MACEs. Feature values are color-coded, with red representing higher values and blue representing lower values. We can see from the SHAP plot that the most important feature is the dose of statins with low values of the dose corresponding to a high risk of MACEs. Interestingly, the VEGFR-2 genotypes appeared to be the fourth top important feature with the risk T-allele corresponding to higher risk of MACEs. The other important features are comorbidity index, coronary artery lesions, LV parameters, such as lateral LV wall, CABG or PCI, body mass index (BMI), and sex (see Figure 3).

Additionally, we constructed partial dependence plots (PDP) and individual conditional expectation (ICE) plots for key features to provide a more intuitive understanding of their impact on predictions (Supplementary Figure S3). PDP shows the average effect of a feature on the predicted outcome, whereas ICE plots illustrate the impact of a feature at the individual observation level, highlighting variability across different data points. The provided PDP and ICE curves offer a detailed visualization of how individual features influence the predicted risk of MACEs in MI patients. PDP graphs depict the marginal effect of each feature, whereas the ICE curves illustrate the variability in predictions across different patients.

For continuous variables, the plots revealed the following trends: an increased BMI and a higher comorbidity index are associated with a greater risk, while a higher statin dosage is linked to a reduced risk. Similarly, greater severity in coronary artery lesions and left main coronary artery lesions leads to an increased risk. The average effect is represented by the orange dashed line, and individual patient trajectories are shown in blue (Supplementary Figure S3).

For categorical features, the bar plots highlight distinct differences in risk levels: male sex, undergoing PCI instead of CABG, lateral LV wall involvement in MI, and the presence of the risk T-allele in the VEGFR2 genotype—all correspond to a greater risk. These visualizations underscore the importance of considering both average trends and individual variances in the assessment of ML model predictive power.

We built the ML model based on only two features: the most important feature—a dose of statins and the genetics—VEGFR2 genotype, and the model performance reached 0.80 ROC AUC, which is only 0.013 less than the best model (Figure 1B). This means that these two predictors can serve as markers in assessing the risk of MACEs in MI patients.

### 3.5 Model risk assessment for individual patients

**Figure 4:**
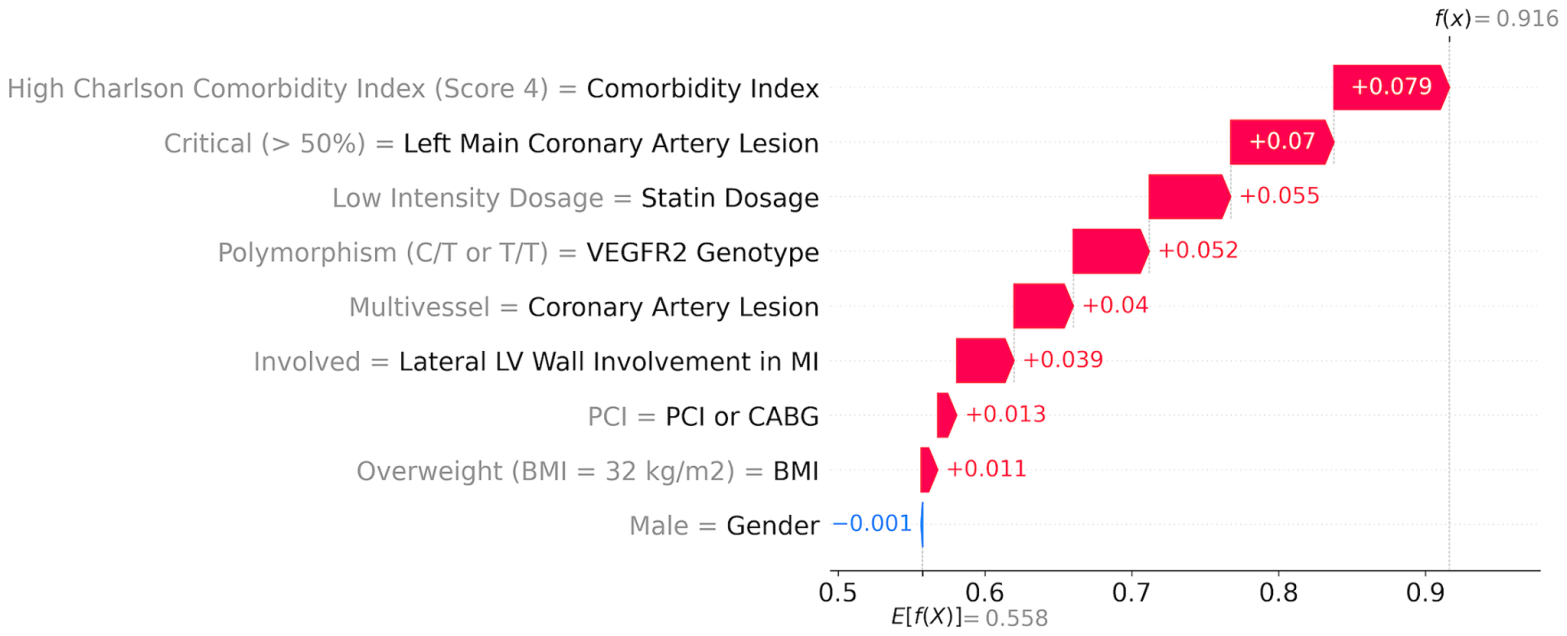
ML model risk assessment for a high-risk patient.

**Figure 5:**
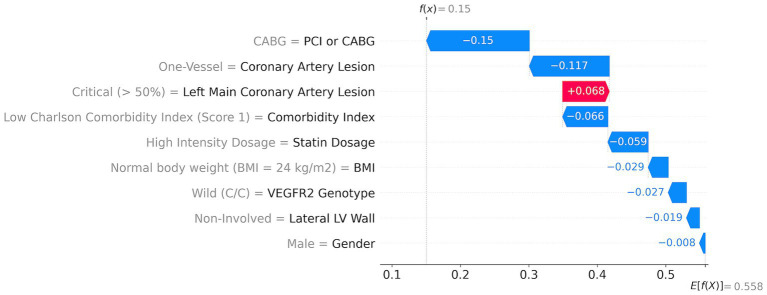
ML model risk assessment for a low-risk patient.

With SHAP methods, one can evaluate the contributions of each parameter to the individual risk of a patient. The ML model risk assessment for high- and low-risk patients with corresponding SHAP values is presented in Figures 4, 5.

A high-risk patient with a 92% risk (Figure 4) has the following top five major contributors: high comorbidity index (score = 4; red arrow in Figure 4) with a 7.9% contribution, followed by the critical left main coronary artery lesion (>50%) with a 7% contribution, low dosage of statins with a 5.5% contribution, risk T-allele with a 5.2% contribution, and multivessel coronary artery lesion with a 4% contribution. Additionally, lateral LV wall involvement, PCI, and a BMI of 32 kg/m^2^ contribute to the risk by 3.9, 1.3, and 1.1%, respectively.

An example of a patient with a low risk of MACEs among the investigated patients (15% risk) shows that all features contribute positively (indicated by blue arrows) to reducing the total risk (Figure 5). The first risk-reducing parameter is CABG followed by the absence of coronary artery lesion. However, left coronary artery lesions increase the risk by almost 7% (indicated by the red arrow in Figure 5). A low comorbidity index and high statin dosage reduce the risk by 6.6 and 5.9%, respectively. Other factors such as normal body weight, non-risk genetics, non-involved lateral LV walk, and sex further reduce the risk cumulatively by 8%.

## 4 Discussion

Several studies have shown strong associations between VEGFs, VEGFRs serum levels, and other growth factors with clinical characteristics and prognosis in ACS (30). VEGFR-2 SNPs have previously been shown to be associated with the development of CVDs such as CAD, ACS, and Kawasaki disease (11) and have also been evaluated as prognostic markers in stroke and ACS. Thus, in the meta-analysis of Qui et al. (10), VEGFR-2 rs2305948 and rs1870377 (both found in exon regions of VEGF receptor-2 and lead to aminoacidic substitutions that reduce the binding affinity of VEGF to VEGF receptor-2), but not rs2071559, were associated with an increased risk of stroke. In another meta-analysis, Wang et al. (31) proved that VEGFR2 polymorphisms (rs1870377, rs2071559, and rs2305948) could be used to identify individuals with increased susceptibility to atherosclerotic cardiovascular disease. In another study, based on the Nanjing Stroke Registry, rs1870377 could predict the 3-month outcome of patients with large artery atherosclerotic stroke (32). Marks et al. showed that none of the VEGFR-2 SNPs (rs1870377, rs2071559, and rs2305948) were associated with mortality in 2067 ACS patients of the New Zealand Coronary Disease Cohort Study (14). We, for the first time, included the VEGFR-2 genotype as a feature in the prognostic model for MI patients’ outcomes and found that the genetic factor is included in the top five most important features.

We also evaluated the impact of various factors—clinical, imaging, genetic, and treatment-related—on risk prognosis. Using the developed ML approach, we demonstrated a sufficient negative impact of various factors on combined clinical endpoints, including cardiovascular death, recurrent ACS, stroke, and myocardial revascularization. The key factors are as follows: clinical factors—male sex, high body mass index, and complex comorbidity; imaging factors—multivessel coronary lesions and LV lateral wall involvement; genetic factors—minor allelic variants of VEGFR-2 rs2305948; and treatment factors—PCI only for the infarct-related coronary artery instead of CABG in cases of multivessel coronary artery disease, and low-intensity statin dosage. At the same time, high versus low doses of statins and CABG versus PCI in the study cohort of MI patients showed significant benefits in reducing MACEs during the 9-year follow-up.

Advanced age, female sex at menopause (33), and comorbidities such as diabetes, obesity, and dyslipidemia are well-known conventional risk factors for MACEs (recurrent ACS, stroke, and death) in MI patients. A dramatic shift has been observed over the last 25 years in the epidemiology of cardiovascular events. The incidence of MI and ischemic stroke has decreased 3- to 4-fold, which can be attributed to major changes in population risk factors with substantial decreases in systolic blood pressure, cholesterol concentrations, and smoking rates, although this has been offset by increases in body mass index, diabetes, and other comorbidities prevalence. Additionally, a positive impact has been made by the wider post-MI use of cardioprotective medications, such as high-dose statins, P2Y12-platelet inhibitors, beta-blockers, and newer drugs in real clinical practice (34–37). Involvement of the lateral LV wall in MI is a negative prognostic marker of maladaptive cardiac remodeling, often requiring more advanced treatment options (e.g., surgical ventricular restoration) to prevent the development of congestive heart failure (38). During the last two decades, the role of multivessel cardiac disease (including the left main coronary artery lesions) has been actively discussed in the prognosis of MI patients (39, 40). According to data from the SWEDEHEART registry, CABG in patients with LMCA disease is associated with lower mortality and fewer MACEs compared to PCI (41). Complete PCI revascularization following MI reduces all-cause mortality, cardiovascular mortality, recurrent ACS, and repeat symptom-driven revascularization. Immediate complete PCI or CABG revascularization in MI may be equally beneficial, but it requires additional head-to-head trials (42).

Comparison to other ML prognostic models revealed similarities in our findings with respect to clinical and imaging factors. In the study (15), feature importance analysis revealed LVEF, age, diabetes, estimated GFR, multivessel disease, peripheral artery disease, complete revascularization, hemoglobin level, previous bleeding, malignancy, ACE inhibitors or ARB at discharge, and statin therapy at discharge as the most important features to predict all-cause death and recurrent MI. Similar to our results, significant risk factors of MACEs included comorbidities (age, multivessel disease, diabetes, chronic kidney disease, and malignancies), complete myocardial revascularization, and in-hospital and post-discharge high-intensity statin usage.

ML models for predicting all-cause death at 3-year follow-up, based on clinical data from the nationwide perspective registry of AMI in Korea (*n* = 13,104) (43), identified the following top 10 predictors: statin use at discharge, sex, body mass index, use of glycoprotein IIb/IIIa inhibitors, in-hospital duration, coronary lesion classification, NT-proBNP, total cholesterol, door-to-balloon time, and peak troponin I. The majority of the predictors were also shown as significant for the Russian MI population in our prognostic model for the combined end-point. Specifically, the top important features include: coronary lesions, (high-intensity dose) statin use at discharge, sex, and body mass index. Wang et al. (44) presented the ML risk model including 21 patient characteristic variables for a 6-month prediction of MACEs for 1,004 Chinese patients who had undergone coronary revascularization. The model also found age and CABG as the top important factors.

In another large prospective study (45) based on the UK Biobank cohort, the authors revealed 10 predictors for 473,611 CVD-free participants, namely age, sex, cholesterol and blood pressure medications, cholesterol ratio (total/high-density lipoprotein), systolic blood pressure, previous angina or heart disease, number of medications taken, cystatin C, chest pain, and pack-years of smoking. Direct comparison is difficult due to different cohorts (CVD-free participants or post-MI patients); however, cholesterol medications, age, coronary artery disease, and comorbidities (such as diabetes) were also detected as top important factors.

As for the best model performance, the initial model selection was guided by an AutoML framework (FLAML) that evaluated multiple algorithms, including Random Forest, CatBoost, and LightGBM. The AutoML process identified CatBoost as the optimal base estimator, highlighting its balance between accuracy and generalization. Although Random Forest showed higher performance metrics on the test dataset, this can be attributed to overfitting as indicated by its exceptionally high results. CatBoost, on the other hand, demonstrated performance without overfitting, making it a more reliable choice for our clinical application. Boosting algorithms, in general, are well-documented for providing state-of-the-art performance for tabular data. In particular, CatBoost with hyperparameter tuning has been shown to be the best-performing model on average as detailed in (46). This consistency in performance underscores the reliability of CatBoost for our study. Ultimately, CatBoost showed superior performance on the test dataset, reinforcing its suitability as the primary algorithm.

The present study demonstrates that VEGFR-2 genotypes can be used as a predictor of MACEs. While these findings might be specific to the study population due to the lack of external validation, the results provide a solid foundation for conducting larger studies. Such research could lead to the development of a universal prognostic tool for MACEs using this genetic biomarker.

To our knowledge, genetic factors have not previously been used in ML models for predicting MACEs in MI patients, mostly due to the fact that sequencing or even single nucleotide genotyping is not routinely performed in hospitals. In this respect, this study is pioneering in showing that the risk allele of the VEGFR-2 variant is among the top 5 most important factors for predicting long-term outcomes in MI patients.

We see a large potential for integration of the model into current clinical practice. Patient MACE risk assessment can be integrated into telemedicine service and automatically assessed based on patients’ clinical and genetic data. Telemedicine service will provide feedback on patient status and facilitate corrections to the model, thereby improving the prognostic accuracy of the model.

Directions for future research include the improvement of the ML model by training on a larger dataset, performing external validation, and investigating other genetic markers known to be associated with the risk of MACEs. The latter is the subject of extensive research worldwide, and patient genetic information will help to improve personalized treatment strategies.

## 5 Conclusion

We developed an ML model that can predict long-term (9 years) ischemic outcomes in MI patients with ROC AUC of 0.813, based on nine features selected from an initial set of 39. For the first time, we included genetic factors in the ML predictive model for assessing the risk of MI patients from a long-term perspective. VEGFR-2 rs2305948 (C/C, C/T, T/T) genotypes showed high predictive power with risk T-allele increasing the risk of MACEs. Additionally, we demonstrated that high-dose statin therapy for 12 months post-MI, along with other factors, can minimize cardiac risk for patients carrying the risk T-allele.

Integration of VEGFR-2 genotypes in MACE prediction models requires a larger study. However, we find this model suitable for the risk assessment for patients at the Surgut District Cardiology Clinic. Immediate integration of this model into clinical practice requires an easy-to-use risk assessment application.

## Data Availability

The raw data supporting the conclusions of this article will be made available by the authors, without undue reservation.
