# Supplementary material for: Machine learning models for predicting risks of MACEs for myocardial infarction patients with different VEGFR2 genotypes
Source: Front Med (Lausanne). 2024 Sep 5;11:1452239. doi: 10.3389/fmed.2024.1452239 (PMC11410707; doi:10.3389/fmed.2024.1452239)
Supplement: Supplementary file 1 [file Data_Sheet_1.pdf]

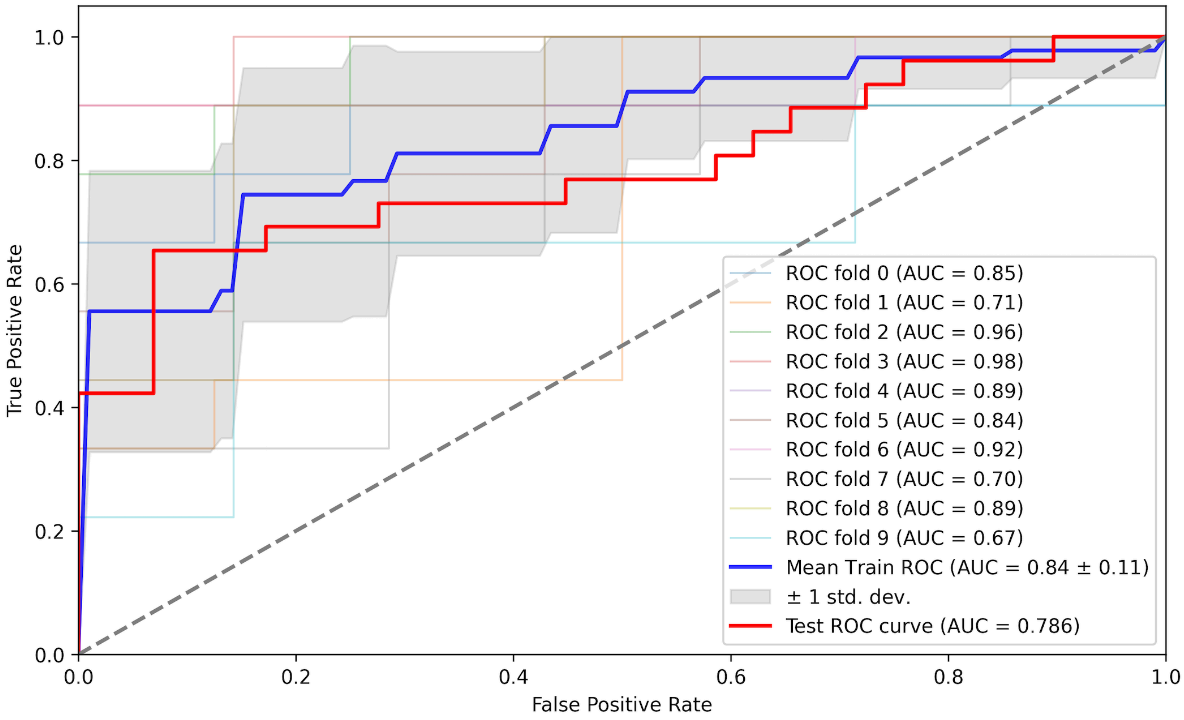

Supplementary Figure 1. ROC Curves for training and test sets for the best CatBoost model.

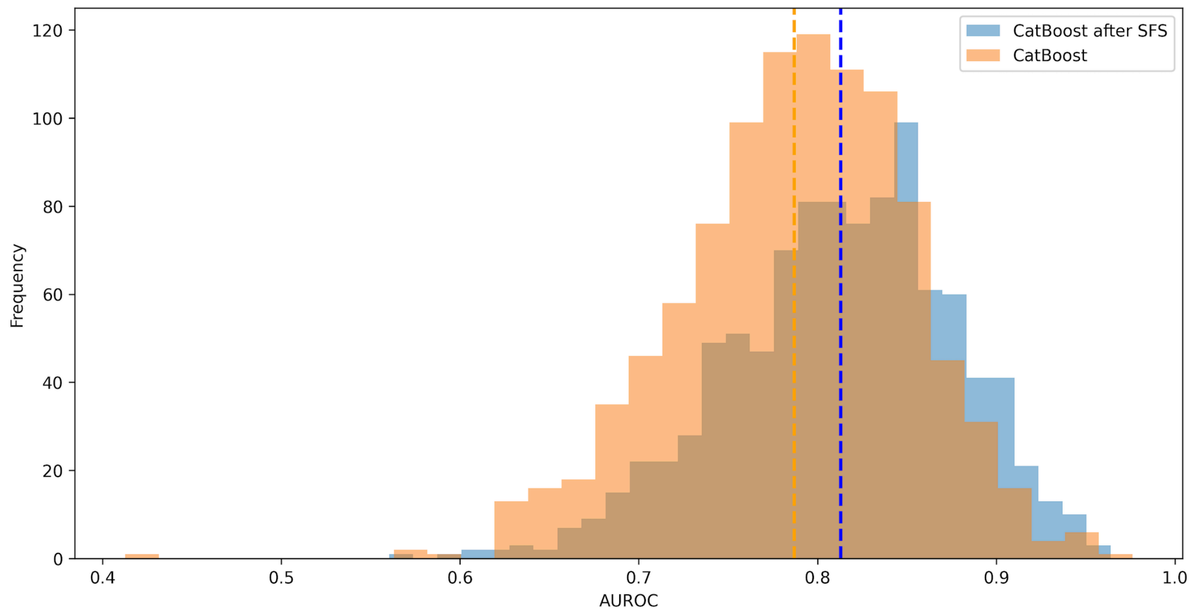

Supplementary Figure 2. Distribution of ROC AUC Scores on bootstrap samples for CatBoost model before (39 features) and after (9 feature) SFS method. T-test method showed statistically significant enrichment (p=5e-19).

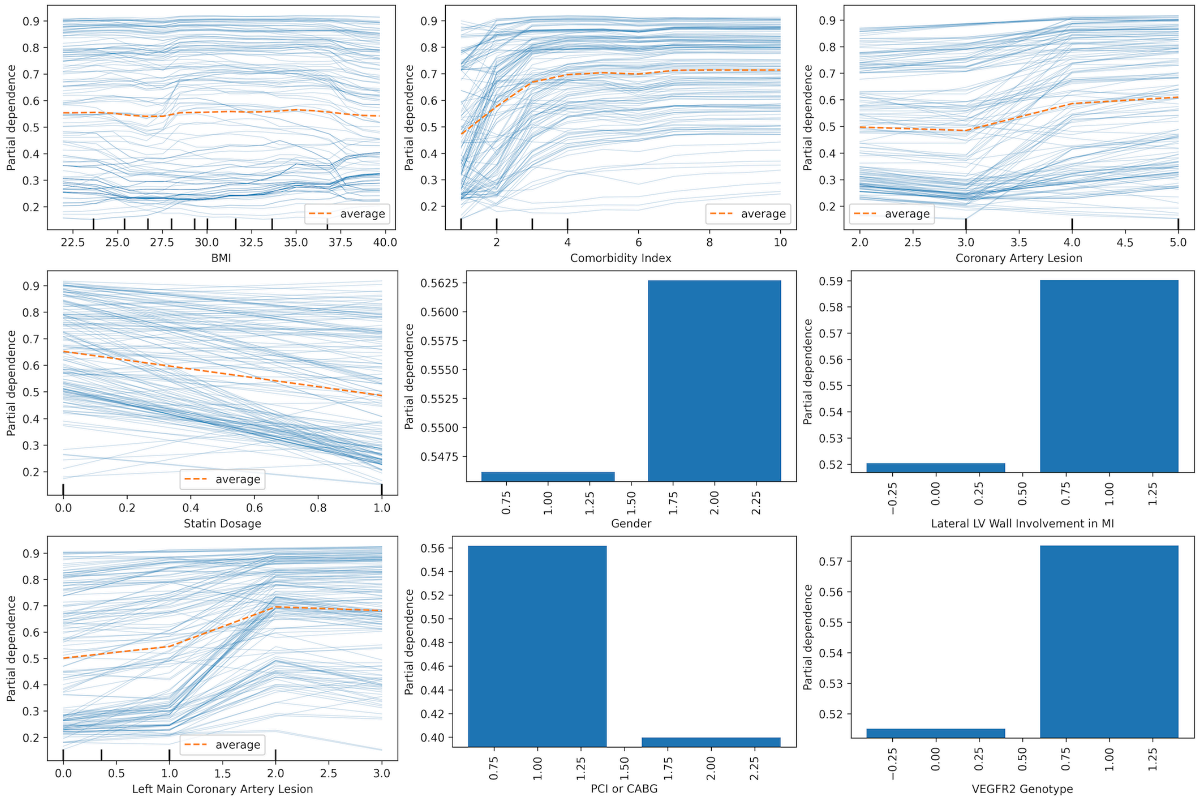

Supplementary Figure 3. Partial Dependence Plots (PDP) and Individual Conditional Expectation (ICE) plots for 9 SFS selected features.
