# Supplementary material for: Machine learning models for predicting risks of MACEs for myocardial infarction patients with different VEGFR2 genotypes
Source: Front Med (Lausanne). 2024 Sep 5;11:1452239. doi: 10.3389/fmed.2024.1452239 (PMC11410707; doi:10.3389/fmed.2024.1452239)
Supplement: Supplementary file 2 [file Table_1.DOCX]

**Supplementary Table 1.**  Description of 9 SFS selected features with corresponding encoded values.

| **Variable** | **Encoded Value** | **Description** |
| --- | --- | --- |
| **BMI** | Numerical values | Body mass index, a measure of body fat based on height and weight (body surface square). |
| **Comorbidity Index** | Numerical values | A score representing the number and severity of comorbid diseases / conditions. |
| **Coronary Artery Lesion** | 1 | No atherosclerotic lesion |
|  | 2 | Non-significant lesion (< 70%) |
|  | 3 | One vessel (lesion ≥ 70%) |
|  | 4 | Two vessels (lesion ≥ 70%) |
|  | 5 | Three or more vessels (lesion ≥ 70%) |
| **Statin Dosage** | Numerical values | Dosage of statins prescribed to the patient. |
| **Gender** | 1 | Male |
|  | 2 | Female |
| **Lateral LV Wall Involvement** | 0 | Not involved |
|  | 1 | Involved |
| **Left Main Coronary Artery Lesion** | 1 | No atherosclerotic lesion |
|  | 2 | Non-significant lesion (< 50%) |
|  | 3 | Significant lesion (lesion ≥ 50%) |
| **PCI or CABG** | 1 | PCI (Percutaneous Coronary Intervention) |
|  | 2 | CABG (Coronary Artery Bypass Grafting) |
| **VEGFR2 Genotype** | 0 | Wild type |
|  | 1 | Alleles with atherothrombotic risk |
